# Effects of Laser Shock Processing on the Mechanical Properties of 6061-T6 Aluminium Alloy Using Nanosecond and Picosecond Laser Pulses

**DOI:** 10.3390/ma18204649

**Published:** 2025-10-10

**Authors:** Martha Guadalupe Arredondo Bravo, Gilberto Gomez-Rosas, Miguel Morales, David Munoz-Martin, Juan Jose Moreno-Labella, Jose Manuel Lopez Lopez, Jose Guadalupe Quiñones Galvan, Carlos Rubio-Gonzalez, Francisco Javier Casillas Rodriguez, Carlos Molpeceres

**Affiliations:** 1 Centro Universitario de Ciencias Exactas e Ingenierías, Universidad de Guadalajara, Blvd. Marcelino García Barragán 1421, esq. Calzada Olímpica, Guadalajara 44430, Jalisco, Mexico; martha.arredondo@academicos.udg.mx (M.G.A.B.); jose.quinones@academicos.udg.mx (J.G.Q.G.); 2 Centro Laser, Universidad Politécnica de Madrid, C/Alan Turing 1, 28038 Madrid, Spain; miguel.morales@upm.es (M.M.); david.munoz@upm.es (D.M.-M.); juanjose.moreno.labella@upm.es (J.J.M.-L.); josemanuel.lopezl@upm.es (J.M.L.L.); carlos.molpeceres@upm.es (C.M.); 3 Escuela de Ingenieria y Ciencias, Tecnologico de Monterrey, Epigmenio González 500 Fracc, San Pablo 76130, Querétaro, Mexico; crubio@cidesi.edu.mx; 4 Centro Universitario de los Lagos, Universidad de Guadalajara, Av. Enrique Díaz de León 1144, Colonia Paseos de la Montaña, Lagos de Moreno 47460, Jalisco, Mexico; francisco.casillas@academicos.udg.mx

**Keywords:** laser shock processing, aluminium 6061-T6, pulsed lasers, compressive residual stress, microhardness

## Abstract

Laser shock processing (LSP) is a surface treatment technique used to enhance mechanical properties such as hardness, corrosion resistance, and wear resistance. This study investigates the effects of LSP on a 6061-T6 aluminium alloy using four treatment conditions: nanosecond (ns-LSP), picosecond (ps-LSP), and a combination of nanosecond–picosecond (nsps-LSP) and picosecond–nanosecond (psns-LSP) pulses. Two laser systems were employed: a Q-switched Nd:YAG laser (850 mJ/pulse, 6 ns, 1064 nm, 10 Hz), and an Ekspla Atlantic 355-60 laser (0.110 mJ/pulse, 13 ps, 1064 nm, 1 kHz). All treatments induced compressive residual stresses up to 1 mm in depth. Additionally, improvements in microhardness were observed, particularly at deeper layers in the combined nsps-LSP treatment. Surface roughness was measured and compared. Among all configurations, the nsps-LSP treatment produced the highest compressive residual stresses (−428 MPa) and greater microhardness at depth. These results suggest that the combined nsps-LSP treatment represents a promising approach to enhance the mechanical performance of metallic components.

## 1. Introduction

Aluminium alloy 6061-T6 is widely used in various industries that require forming processes, especially extrusion, such as orthopaedic devices, turbine blades, rods, and gears, among other components that need low weight, corrosion resistance, hardness, and wear resistance [1,2]. Due to exposure to wear processes, aluminium materials suffer deterioration in their mechanical properties, such as fatigue resistance, wear resistance, and hardness. Over time, different techniques have been investigated to improve these properties, including laser shock peening (LSP) and conventional shock peening (SP).

Since 1962, Askaryan and Moroz studied the effect of the pressure exerted on the surface by a laser pulse. In 1974 Fairand and Mallozzi obtained the first patent for laser shock treatment (LSP). This innovative surface treatment technique improved the mechanical properties of materials by generating residual compressive stresses on the treated surface [3,4,5,6]. This process generates shock waves that help to improve mechanical properties such as fatigue, corrosion, and wear resistance [7], in addition to achieving a better surface finish compared to other surface treatments.

In previous works, Gómez-Rosas et al. (2010) [8] and He et al. (2021) [9] have shown that traditional nanosecond LSP (ns-LSP) surface treatment improves the mechanical properties of aluminium alloys. High compressive residual stress values extending to depths close to 1 mm were reported in [8]. Petronic et al. (2017) [10] applied picosecond LSP (ps-LSP) to a Nimonic 263 alloy, demonstrating an increase in microhardness near the surface, which gradually decreases with depth up to approximately 1 mm. They concluded that this is a good alternative for improving the mechanical properties of materials [10]. Keshavarz et al. (2016) induced residual stresses in a silicon biotemplate using an ultrashort pulse laser (USPL) in the femtosecond range, which caused silicon recrystallization [11]. In 2020, Rujian Sun et al. compared combined nano and femtosecond LSP treatments and found that the hardness at the nano-depth was higher in the femto-nanosecond combination, which they attributed to better laser absorption and strong plastic deformation [12]. In 2020, Loja, and in 2022, Lopez J., performed laser peen forming with a picosecond laser and achieved curved deformations, avoided wear, and induced residual compressive stresses [13,14]. Recently, Hu et al. (2025) showed the corrosion behaviour of laser powder bed fusion TA2-Cu-Q345 composite plate subjected to picosecond LSP, obtaining improved corrosion resistance in the treated samples [15].

Since each LSP pulse covers a small area, pulses are overlapped and scanned in a zigzag pattern to ensure full coverage. However, this induces residual stress anisotropy, leading to variations in stress magnitude between the x- and y-directions on the surface. This effect has been observed experimentally [8,16] and predicted in numerical simulations [16,17]. Additionally, Correa et al. (2015) demonstrated through simulations of LSP treatments that using random scanning patterns significantly reduces residual stress anisotropy, compared to the conventional LSP zigzag pattern [17].

**Figure 1:**
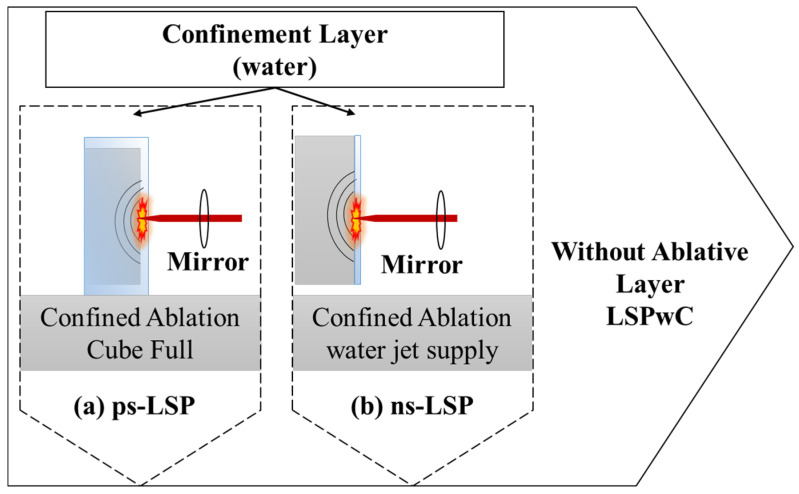
Configuration of the two distinct LSP treatment modalities with confined ablation: (**a**) Cube Full configuration, (**b**) water jet supply.

LSP is based on the interaction between laser and matter. This phenomenon begins when a high-energy pulsed laser beam strikes the surface of the material; the beam is concentrated with a lens, resulting in a large amount of energy per unit area, vaporising part of the material, and simultaneously generating a plasma (ionised gas) that expands rapidly. The expansion of the plasma occurs in the opposite direction to the incidence of the laser pulse, creating a pressure of the order of GPa on the surface of the material. This creates a shock wave that propagates through the bulk, causing both elastic and plastic deformations, which contributes to the generation of stresses in the same direction of wave propagation, as shown in Figure 1. LSP treatments without a sacrificial layer (LSPwC) were used in this work [18].

In this study, 6061-T6 aluminium alloy samples were used, the LSP treatment was performed with picosecond and nanosecond lasers and combinations of both, and residual stresses were analysed before and after treatment. A wavelength of 1064 nm was used for both treatments. When combining treatments, the same scan direction was used for both processes. After treatment, residual stress tests using the hole-drilling method and microhardness measurements using a Vickers indenter and contact profilometry were performed to determine the roughness of the material.

## 2. Materials and Methods

### 2.1. Preparation of Samples Before Treatment

The 6061-T6 aluminium alloy target was used; this contains 96–99% aluminium and the rest of the elements that compose the alloy are shown in Table 1 [13].

The sheet has a thickness of 7 mm and was cut into targets with dimensions of 50 mm × 50 mm. A polishing process was performed on the face to be treated, until a semi-mirror finish was obtained. The objective of this process is to eliminate scratches and to have homogeneity in the surface to be treated.

### 2.2. Experimental Setups and Mechanical Characterizations

Two different experimental arrangements were used: one with water jet supply to the surface to be treated for the ns-LSP treatment, and the other experimental arrangement was with a confined ablation in cube full for the ps-LSP treatment. Both arrangements are shown in Figure 1.

**Figure 2:**
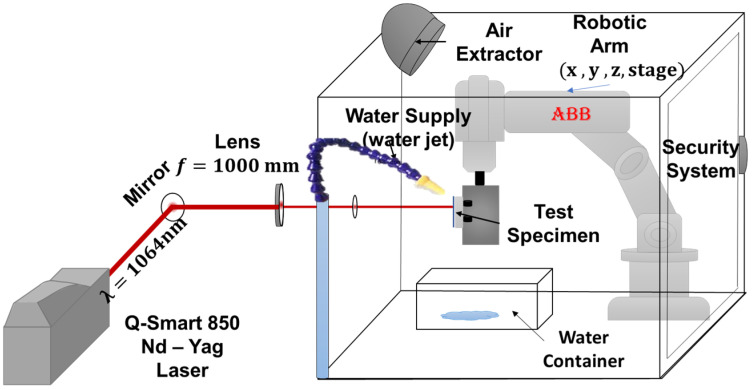
Experimental setup for ns-LSP surface treatment.

Two different types of pulsed lasers were used; for the ns laser (Figure 2), a Nd-YAG Quantel Q-Smart 850 laser was used, emitting in nanosecond (ns) by a flash-lamp, with a repetition rate of 10 Hz, energy output of 850 mJ, and a beam output diameter of 9 mm. A lens with a focal length of f = 1000 mm was used to obtain a spot diameter of 1 mm to concentrate the energy and obtain GW/cm^2^ power densities. The parameters used for the ns-LSP treatment are shown in Table 2. The movement was performed by means of a robotic arm model IRB-120 ABB, with (x,y,z) movements.

**Figure 3:**
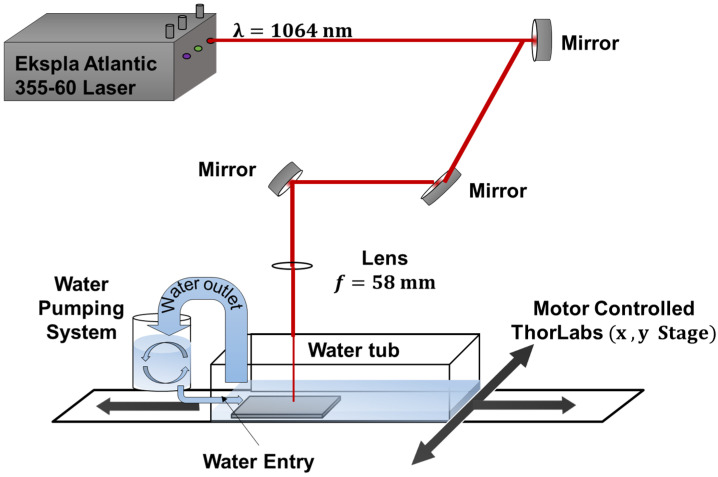
Experimental setup for ps-LSP surface treatment.

For the ps laser, an Ekspla Atlantic 355-60 laser was used, with repetition rates of 1–20 × 10^3^ Hz, energy output of 0.110 mJ, and a spot diameter of 4.9 × 10^−3^ cm. Both lasers operated at a wavelength of 1064 nm. The experimental setup was used with the full cube configuration, as can be seen in Figure 3. The optical path is composed of mirrors to direct the beam towards a lens with a focal length of 58 mm; when passing through the lens, the energy is concentrated to generate power densities of GW/cm^2^, and to irradiate the piece. To perform the movement of the target, a system of x,y motors was used. The water was kept in a continuous rotation with a pump, to avoid the accumulation of residues in the water of the tank. The laser parameters used to perform the treatment can be seen in Table 2.

To ensure high energy transmission in both systems, the optical components are coated with an anti-reflection coating (AR) suitable for the wavelength used.

The treated area with ns-LSP treatment was 25 mm × 25 mm, with a pulse density of 2500 pulses/ cm^2^. We worked with a spot size of 1 mm diameter and an energy of ~0.725 J. The power density obtained was 1.2 × 10^10^ W/cm^2^.

For the ps-LSP experiment, the treatment area was 20 mm × 20 mm. Pulse density were used, 1 × 10^7^ and 2 × 10^6^ pulses/cm^2^ were used. The spot size had a diameter of 4.80 × 10^−3^ cm, with an energy per pulse of 1.15 × 10^−4^ J. This results in a power density of 4.89 × 10^11^ W/cm^2^.

**Figure 4:**
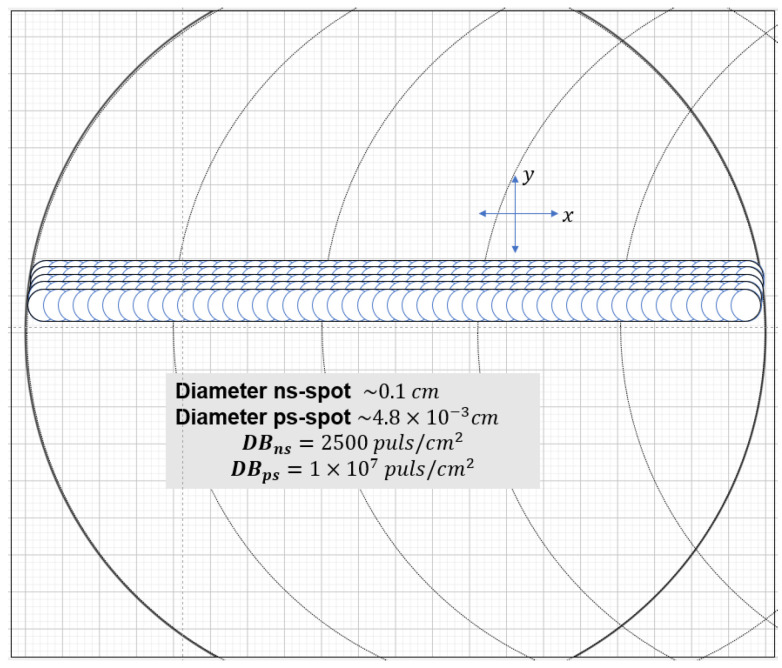
Comparation of laser spot size between ns-LSP and ps-LSP.

Treatment was also performed by combining both sweeps of the two systems, combining the nano–pico (nsps-LSP) and pico–nano (psns-LSP) treatment. In Figure 4, we can see the comparison of laser spot size between ns-LSP and ps-LSP. A total of four different treatments were performed, as shown in Table 3.

The residual stress (RS) was determined using the hole drilling technique. The equipment used for this procedure was the Vishay RS-200 (Milling Guide, Wendell, NC, USA) and the model CEA-06-062UL-120 strain gauge, which was mounted in the same direction as the LSP scan. The parameters used to determine the residual stresses were Young’s modulus (E = 68.9 GPa) and Poisson’s ratio (μ = 0.33). A mathematical algorithm based on ASTM E837-01 [19,20,21] was used to determine the residual stresses.

**Figure 5:**
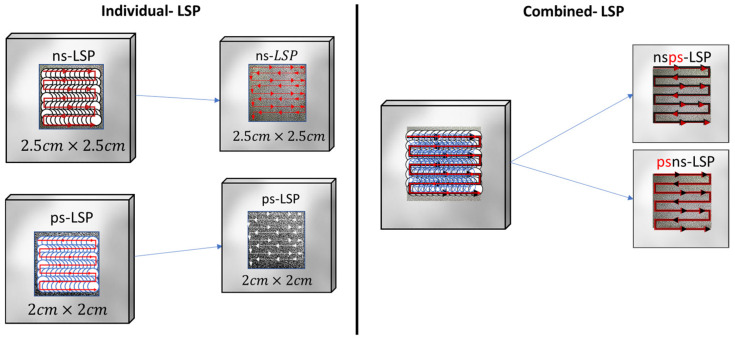
Graphical representation of the individual and combined treatments.

In Figure 5, we can see a graphical representation of the sweep direction of the individual treatments in the same direction for both cases. In the combined treatments, the black arrow indicates the sweep direction of the ns-LSP treatment and the red arrows indicate the sweep direction of the ps-LSP treatment, both in the same direction.

The evaluation of depth microhardness was performed using a MXT30 microhardness tester,(Matsuzawa Co., Ltd., Akita, Japan) following the specifications of ASTM E384. For depth characterisation, the samples were sectioned transversely, and 15 measurements were taken at each depth level, with reference positions set at $Y_{0}=0\;mm$, followed by $Y_{1}=0.25\;mm$, $Y_{2}=0.50\;mm$, $Y_{3}=0.75\;mm$, and $Y_{4}=1.00\;mm$. A total of 15 measurements were performed on each sample.

Finally, roughness tests were performed using a surface contact profilometer with DEKTAK 150 equipment (Veeco Instruments Inc., Plainview, NY, USA). The parameters used for the measurements were a load of 4 mg, a resolution of 0.056 μm, a measurement range of 6.5 μm, and a tip radius of 2.5 μm. Five repetitions per sample were carried out to finally obtain an average roughness (Ra).

## 3. Results

### 3.1. Residual Stresses (RS) Results

**Figure 6:**
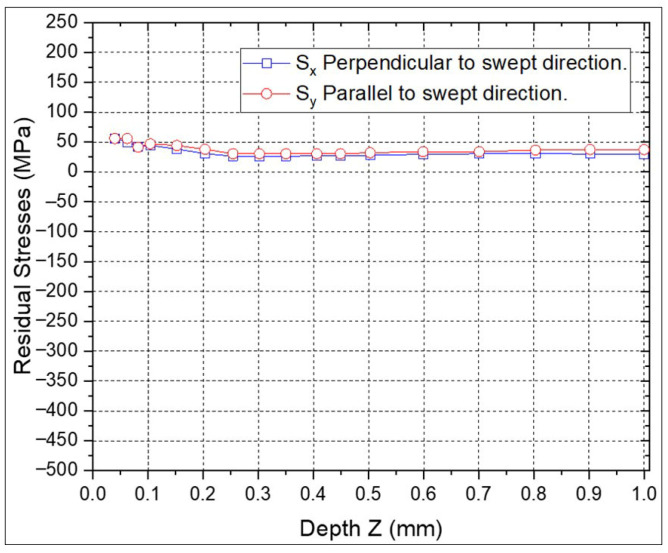
RS on 6061-T6 aluminium alloy target without LSP treatment.

The RS versus depth were measured using the hole drilling method to a depth of 1 mm in all the samples of the Al6061-T6 alloy. In Figure 6, the RS in the sample without LSP treatment is shown, where “Sy” and “Sx” represent the RS, parallel, and perpendicular to the swept direction, respectively. It can be observed that both RS values are tensile, and very similar in value, being between 25 and 60 MPa, and from 0 to 1 mm in depth. These stresses are due to the polishing process prior to the LSP treatment of the 6061-T6 aluminium alloy.

**Figure 7:**
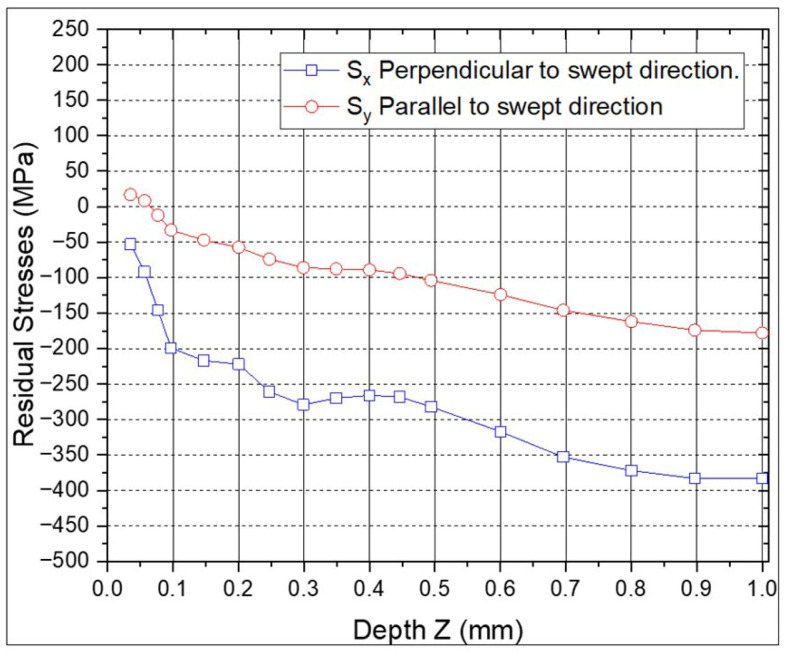
Residual stresses with ns-LSP treatment.

**Figure 8:**
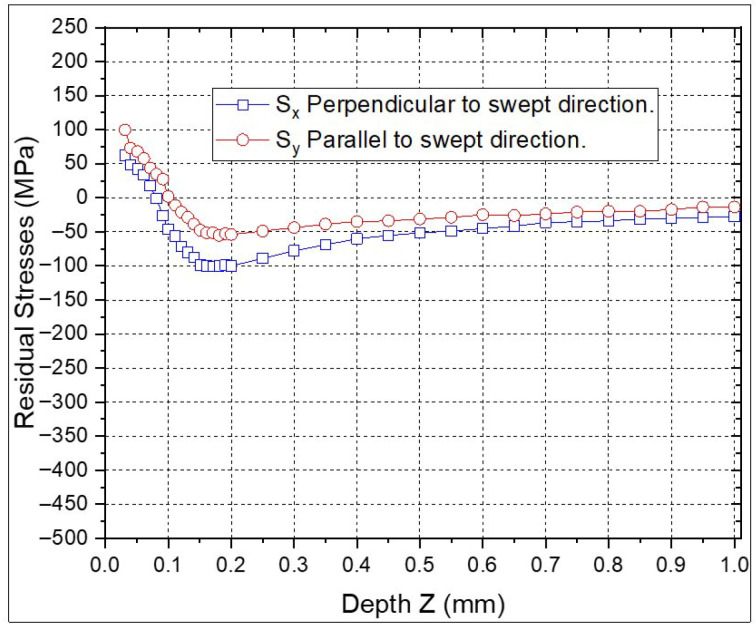
Residual stresses with ps-LSP treatment.

The RS profiles for the individual ns-LSP and ps-LSP treatments are shown in Figure 7 and Figure 8. In the specimen with ns-LSP treatment with a pulse density of 2500 pulses/cm^2^ (Figure 7) the highest compressive residual stresses are found, both in the direction parallel and perpendicular to the sweep. In the first 0.05 mm of depth, in the RS parallel to the scanning direction tensile RS are observed; however, it can be observed that at a depth between 0.8 and 1.0 mm, compressive RS between −350 and −400 MPa are obtained in the perpendicular direction, and in the parallel direction, between −200 and −150 MPa compressive RS are found at the same depth.

When comparing the ps-LSP treatments (Figure 8), which have a pulse density of 1 × 10^7^ pulses/cm^2^, the highest compressive RS are −100 MPa in the perpendicular direction and −55 MPa for the parallel direction at a depth of 0.18 mm. The RS values in both directions show less variation between points in the ps-LSP treatments compared to the ns-LSP points in the individual treatments.

In the samples with combined treatments, two different sequences were applied: one where the ps-LSP treatment was performed first, followed by ns-LSP, and another where ns-LSP was applied first, followed by ps-LSP. Each combination was tested using the same pulse density for ns-LSP and ps-LSP treatments.

**Figure 9:**
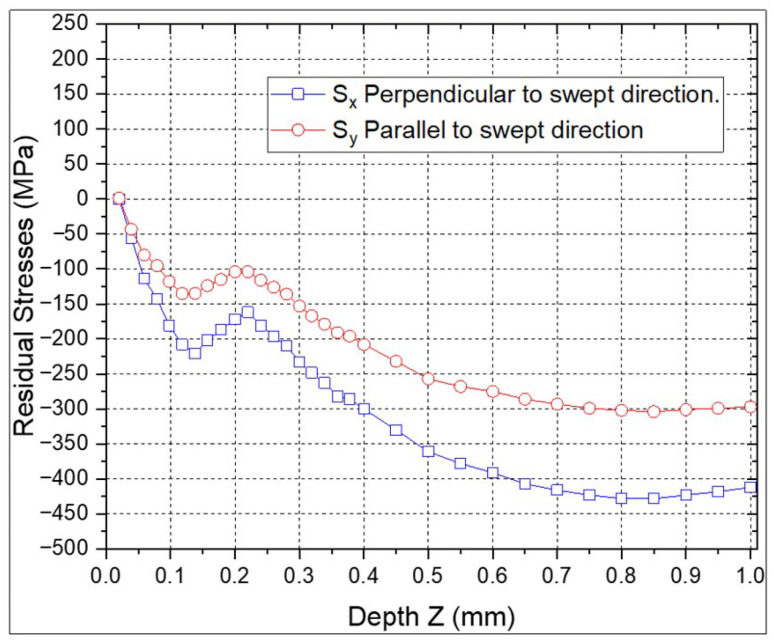
Residual stresses with treatment combination, nsps-LSP.

**Figure 10:**
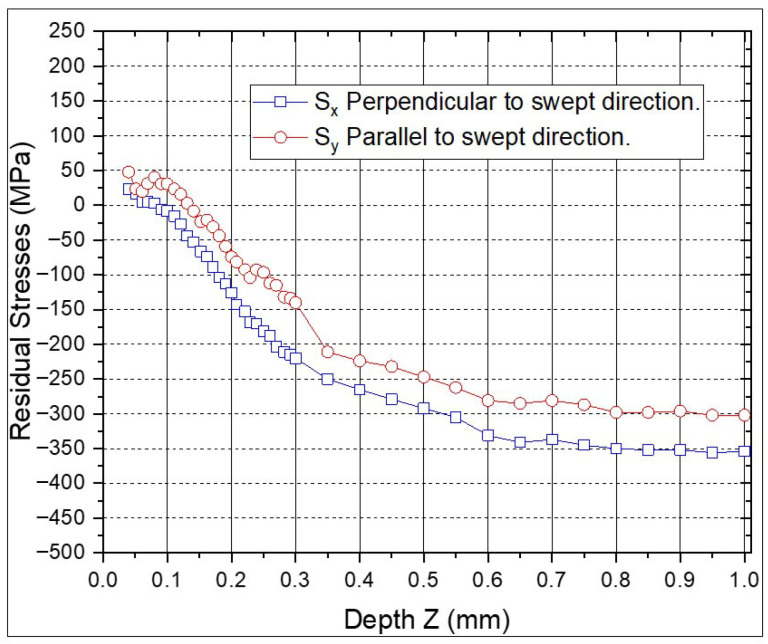
Residual stresses with the psns-LSP treatment combination.

Figure 9 and Figure 10 show the RS values of the combinations with ns-LSP and ps-LSP treatment. The profile shows that the RS value perpendicular to the sweep direction is 50% lower for the psns-LSP combination compared to nsps-LSP. The parallel RS values are similar in both curves.

In Figure 9, the highest compressive residual stresses are observed in the direction perpendicular to the sweep of −428 MPa and for the parallel direction of −304 MPa for the nsps-LSP treatment at a depth of 0.85 mm; the tensile RS are 1.05 MPa at a depth of 0.02 mm and only in the direction parallel to the sweep. For the psns-LSP treatment, shown in Figure 10, the highest compressive RS are −356 MPa in the perpendicular direction and −302 MPa in the parallel direction at a depth of 0.95 mm; tensile RS between 50 and 100 MPa is observed in the first micrometres of depth. Table 4 shows the maximum compressive residual stress values obtained for each treatment as a function of depth.

### 3.2. Microhardness Results

**Figure 11:**
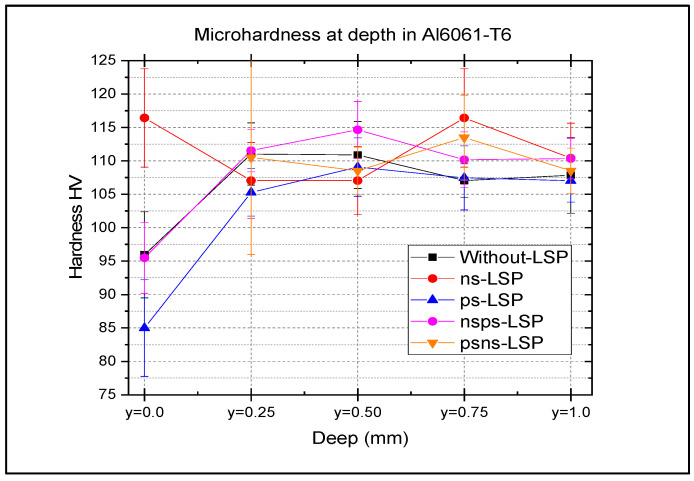
Depth microhardness profile at 500 gF load, comparison of untreated vs. ns-LSP, ps-LSP, nsps-LSP, psns-LSP.

Figure 11 shows the microhardness profiles as a function of depth (up to 1 mm) for the four treatments compared to the untreated sample. The maximum microhardness values with respect to depth are as follows: with ns-LSP, it can be seen that at a depth of 0.75 mm, the microhardness value is ~116 HV. In the sample treated with ps-LSP, there is no increase in microhardness with depth. The microhardness profiles in the nsps-LSP treatment combination show that at a depth of 0.25 mm, the microhardness values are ~115 HV. The microhardness profiles in the psns-LSP treatment combination show that at a depth of 0.25 mm, the microhardness values are ~113 HV.

### 3.3. Roughness

Table 5 shows the roughness profiles by contact profilometer; here it can be observed that the values both parallel and perpendicular to the scanning direction for each of the treatments are very similar. The roughness values after treatment are between 6.3 μm and 12.1 μm, where it can be observed that in ps-LSP the lowest increase is obtained with respect to the base specimen and the highest increase in roughness is found in the specimen treated with psns-LSP of 12.1 μm.

## 4. Discussion

The residual stress values obtained from the ns-LSP treatment are in good agreement with previously reported data, confirming the reliability of our experimental approach [8,19]. In contrast, the ps-LSP treatment led to significantly lower residual stresses. This difference can be attributed to the distinct ablation and interaction mechanisms involved: while the ns regime is predominantly thermally driven, the ps regime combines thermal and electronic processes, which in turn influence the generation and propagation of shock waves.

Regarding the residual stress curves in the parallel and perpendicular directions obtained in the combined treatments (nsps-LSP, psns-LSP), a significant reduction in their separation is observed when compared to the traditional ns-LSP treatment. This behaviour may be attributed to the anisotropy phenomenon reported in [16]. In conventional treatments, the decrease in Sx is typically associated with the tensile stress introduced by the overlap of consecutive laser pulse columns in the x-direction. However, in ps-LSP treatments, the reduced overlap minimises this effect, leading to a modified stress distribution that influences the subsequent ns-LSP treatment. This phenomenon will be further investigated in future work.

In terms of microhardness, the ps-LSP treatment yielded the lowest values, even lower than those of the base material. This behaviour may be explained by the nature of the energy–matter interaction, the high repetition rate, and the surface effects induced by the ultrashort (ps) laser pulses [22,23]. In contrast, the highest microhardness values were obtained with the nsps-LSP treatment at depths of 0.25 mm, 0.50 mm, and 1 mm. This improvement could be related to the initial treatment ps-LSP laser [14], which may produce a thermal effect that enhances energy absorption during the subsequent ns-LSP process. Finally, regarding the surface roughness, the values obtained across all treatments are consistent with those typically observed in LSP processes without a sacrificial layer (LSPwC) [18,19]. For industrial applications, such roughness levels may be acceptable in certain cases; otherwise, they can be improved through a post-treatment polishing process.

## 5. Conclusions

This study experimentally demonstrated that all laser shock peening (LSP) treatments—ns-LSP, ps-LSP, nsps-LSP, and psns-LSP—generate compressive residual stresses up to a depth of 1 mm. In contrast, the untreated sample exhibited residual stress values close to zero. Among all treatments, the combined nsps-LSP process yielded the highest compressive residual stress, reaching −428 MPa at a depth of 0.85 mm. Similarly, the psns-LSP treatment achieved high compressive stress levels of −407 MPa at 0.9 mm, while also reducing the difference between the residual stress curves in parallel and perpendicular directions. This behaviour may be attributed to the initial ps-LSP pre-treatment, which could enhance energy absorption during the subsequent ns-LSP step through a thermal effect [14].

The residual stress values obtained from the ns-LSP treatment are consistent with previously reported values [8]. In contrast, the ps-LSP treatment resulted in significantly lower residual stresses. This outcome is expected due to the different ablation and interaction mechanisms: the ns regime is primarily thermally driven, while the ps regime involves both thermal and electronic interactions, which affect the generation and propagation of shock waves.

Regarding microhardness, the highest surface value was recorded in the ns-LSP treatment (116 HV). However, at deeper layers, the highest microhardness value was obtained with the nsps-LSP treatment at depths of 0.25 mm, 0.50 mm and 1 mm.

As for surface roughness, similar Ra values were found in both scanning directions. The lowest Ra (6.3 μm) was observed in the ps-LSP treatment, while the highest value (12.2 μm) occurred in the psns-LSP specimen.

Overall, this study confirms that nanosecond, picosecond, and combined LSP treatments are effective for inducing compressive residual stresses and modifying surface properties. In particular, the combined nsps-LSP treatment stands out for generating higher compressive stresses and improved microhardness at depth, compared to conventional ns-LSP. This combined treatment could be used to improve the mechanical properties of metallic components in industrial application.

## Figures and Tables

**Table 1:** Chemical composition of 6061-T6 aluminium alloy used in this study [13].

**Chemical Composition**									
Element	Si	Mg	Fe	Cu	Mn	Cr	Zn	Ti	Others
%	0.4–0.8	0.8–1.2	0.7	0.15–0.40	0.8–1.2	0.04–0.35	0.25	0.15	0.15
**Mechanical Properties**									
Heat Treatment	Density	Poisson Coefficient		Tensile Strength	Yield Strength	Elongation (%)		Young Module	Vickers Hardness
T6	g/cm^3^			MPa	MPa	1/16 Thickness	1/2 Diameter	GPa	HV
	2.70	~0.33		~310	~276	~12	~17	68.9	107

**Table 2:** Laser beam parameter for the different ns-LSP and ps-LSP treatments.

Make and Model	Pulse Energy	Pulse Width	Pulse Frequency	Beam Diameter on Sample	Power Density	Pulse Density
	mJ	s	Hz	cm	$W/cm^{2}$	$Pulse\;{cm}^{-2}$
Quantel Q-Smart 850 Nd-YAG	850	$6\times 10^{-9}$	10	0.1	$1.2\times 10^{10}$	2500
Ekspla Atlantic 355-60	0.110	$13\times 10^{-12}$	$1\times 10^{3}$	$4.9\times 10^{-3}$	$4.9\times 10^{11}$	$1\times 10^{7}$

**Table 3:** Nomenclature and description of the different LSP treatments.

Nomenclature	Meaning	Pulse Density (Pulses/cm^2^)	Power Density $W/cm^{2}$
Without-LSP	Targets without LSP treatment		
ns-LSP	Targets treated with ns	2500	$\mathrm{ns}\;:1.2\times 10^{10}$
ps-LSP	Targets treated with ps	$1\times 10^{7}$	$ps\;:4.9\times 10^{11}$
nsps-LSP	Targets treated with ns and post ps	2500 and $1\times 10^{7}$	$\mathrm{ns}\;:1.2\times 10^{10}$ $ps\;:4.9\times 10^{11}$
psns-LSP	Targets treated with ps and post ns	$1\times 10^{7}$ and 2500	$ps\;:4.9\times 10^{11}$ $ns\;:1.2\times 10^{10}$

**Table 4:** Results of the highest compressive residual stress values and their depth for each treatment.

LSP Treatments	Compressive Residual Stresses Perpendicular (Sx) (MPa)	Depth (mm)
ns-LSP	−383	1.0
ps-LSP	−100	0.2
nsps-LSP	−428	0.85
psns-LSP	−356	0.95

**Table 5:** Roughness (Ra) value by contact profilometer.

Target	Roughness (Ra) (μm)	
without	0.334	±0.129
ns-LSP	7.276	±0.585
ps-LSP	6.389	±0.455
nsps-LSP	7.167	±0.539
psns-LSP	12.109	±0.504

## Data Availability

The original contributions presented in this study are included in the article. Further inquiries can be directed to the corresponding author.
